# The Brief Case: Uncommon pathogen, unusual presentation—*Ureaplasma urealyticum* in a post-laminectomy infection

**DOI:** 10.1128/jcm.01857-25

**Published:** 2026-10-14

**Authors:** Ana Collazos Blanco, Juana Alicia Fernández Gonzalez, Tatiana Mata Forte, Mercedes Marín, Sofía Santiño Gómez, Aurora Felipa Molina Foncea, Alicia García Señán, Adrián Nuñez Bello, María Simón Sacristán

**Affiliations:** 1 Department of Clinical Microbiology and Parasitology, Hospital Central de la Defensa “Gómez-Ulla” C.S.V.E. 221908 , Madrid, Spain; 2 Department of Neurosurgery, Hospital Central de la Defensa “Gómez-Ulla” C.S.V.E. 221908 , Madrid, Spain; 3 Department of NRBC-Infectious Diseases and High-Level Isolation Unit, Madrid, Spain; 4 Department of Clinical Microbiology and Parasitology, Hospital General Universitario Gregorio Marañón, Madrid, Spain; 5 Medicine Department, School of Medicine, Universidad Complutense, Madrid, Spain; 6 CIBERES, CIBER de Enfermedades Respiratoias, Instituto Carlos III, Madrid, Spain; 7 Medicine Department, School of Medicine, Universidad de Alcalá, Madrid, Spain; Endeavor Health, Evanston, Illinois, USA

**Keywords:** *Ureaplasma *spp., laminectomy, infection

## CASE

A 75-year-old man with no relevant medical history was admitted after developing acute left ankle pain following a period of prolonged immobility. After multiple courses of nonsteroidal anti-inflammatory drugs with no significant clinical improvement, multilevel spinal canal stenosis was diagnosed. In April 2025, the patient underwent decompressive laminectomy at various levels.

In the postoperative period, the patient developed two episodes of acute urinary retention, both of which required bladder catheterization. Seven days after the second episode, the patient developed hematuria. Although urine sediment analysis showed no evidence of urinary tract infection, the drainage bag was replaced, and empirical antibiotic therapy with cefixime 200 mg every 12 h was initiated for 5 days. No cultures were obtained during this episode.

Early postoperative follow-up revealed a fluctuant subcutaneous swelling adjacent to the surgical wound incision without inflammatory signs. Three weeks later, erythema and localized warmth developed, and computed tomography demonstrated deep and superficial fluid collections.

In view of these findings, urgent surgical exploration with wound revision and surgical debridement was performed. Intraoperatively, macerated and devitalized tissue with a characteristic “cooked” and friable appearance was identified. Liquid samples from the deep and superficial surgical area were processed, and Gram-stained smears (performed using a standard technique with safranin as counterstain) revealed no microorganisms. Furthermore, no growth was observed in conventional culture media (blood, chocolate, and brucella agar) or in enriched brain heart infusion broth for the detection of aerobic and anaerobic bacteria. All of them remained negative after 7 days of incubation at 37°C. Empirical antimicrobial therapy was initiated with extended-infusion cefepime 2 g q8h and daptomycin 850 mg q12h.

One month after surgery, magnetic resonance imaging (MRI) showed persistent fluid collections with minimal radiological improvement, prompting escalation to meropenem 2 g q8h and vancomycin 1 g q12h.

During the 3 weeks following surgical debridement, no leukocytosis or procalcitonin elevation was observed. C-reactive protein (CRP) was mildly elevated (7.21–10.1 mg/dL) (normal range, 0.06–0.5 mg/dL), and the patient experienced intermittent low-grade fever with persistently negative blood cultures.

Interventional radiology performed additional drainage, and two newer liquid samples were submitted. Gram staining showed no microorganisms in either specimen, although polymorphonuclear leukocytes were present in the purulent deep sample. Both specimens were processed for culture as previously described, and no microbial growth was observed after 7 days of incubation at 37°C.

These samples were referred to a reference laboratory for universal 16S rRNA gene polymerase chain reaction (PCR) followed by Sanger sequencing, which yielded a positive result (1). Sequence analysis of the amplicon identified *Ureaplasma* spp., and species-level identification was performed using a specific quantitative real-time polymerase chain reaction (qPCR) assay according to the manufacturer’s instructions (Allplex STI assay), which confirmed the presence of *Ureaplasma urealyticum*. Samples obtained from the first surgical debridement procedure had been preserved at −20°C and were subsequently analyzed by a species-specific PCR assay, confirming the presence of *Ureaplasma urealyticum* in the initial surgical specimens.

Meropenem and vancomycin were discontinued, and targeted therapy with doxycycline 100 mg q12h was initiated.

**Fig 1:**
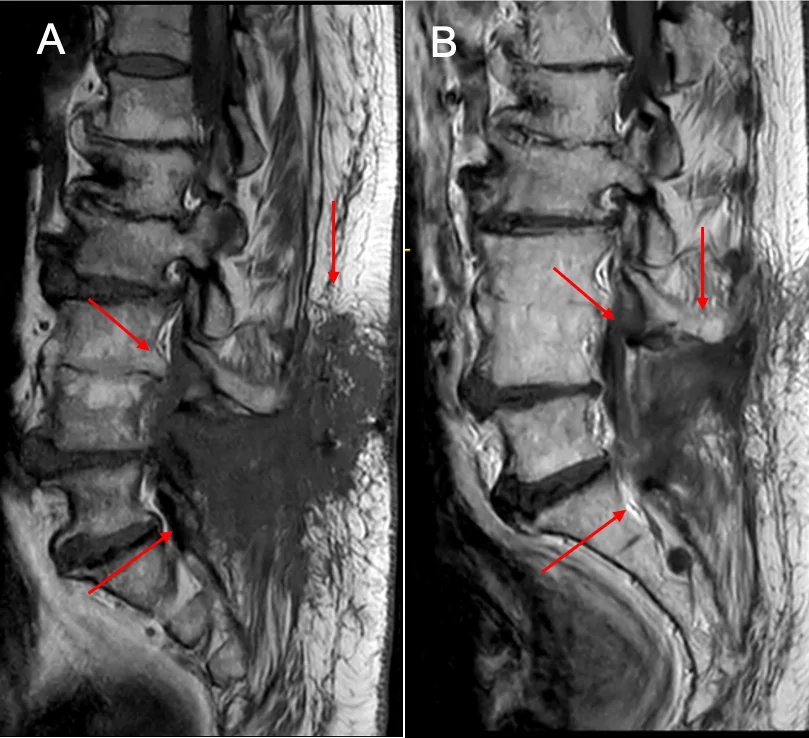
MRI on 23 May 2025 and 17 September 2025. (**A**) Initial MRI showing an irregularly bordered collection suggestive of an abscess, including a deep abscess exerting significant mass effect on the thecal sac at the L4–L5 level (left arrows), and a more superficial subcutaneous abscess (right arrow), with no clear signs of cerebrospinal fluid fistula. In addition, marked edema is observed in the subcutaneous tissue. (**B**) Follow-up MRI performed shows the resolution of the surgical deep abscess and the serohematic collection at the level of the subcutaneous tissue, demonstrating the treatment response.

The patient showed progressive improvement, CRP levels returned to the normal range, and follow-up MRI demonstrated complete resolution (Fig. 1). The total duration of antimicrobial therapy was 8 weeks.

Post-recovery immunological and urological evaluations were unremarkable, with no evidence of underlying immunodeficiency.

## DISCUSSION

*Ureaplasma* spp. belong to the class *Mollicutes* and are characterized as the smallest free-living and self-replicating organisms with a reduced genome, which limits their metabolic pathways and renders them highly dependent on specialized environmental conditions. From a diagnostic and therapeutic perspective, the most critical characteristics of these microorganisms are the absence of a peptidoglycan cell wall and their specific growth requirements, which significantly influence their antimicrobial susceptibility and laboratory diagnosis (2).

Due to the lack of a cell wall, *Ureaplasma* spp. are inherently resistant to beta-lactam antibiotics and glycopeptides, which are commonly used as first-line empirical treatments for most systemic infections (3). This intrinsic resistance frequently explains the initial failure of conventional antibiotic therapy before accurate pathogen identification (4).

The absence of a cell wall prevents these microorganisms from being visualized using conventional Gram staining, creating an important diagnostic challenge. Therefore, alternative visualization methods are required. Among these, acridine orange staining has proven particularly useful for the detection of *Ureaplasma* spp. in blood or other sterile body fluids using fluorescence microscopy. In contrast, other conventional stains such as Dienes, Giemsa, and May–Grünwald–Giemsa demonstrate limited diagnostic utility due to the small size and pleomorphic nature of *Ureaplasma* species cells.

Successful culture of *Ureaplasma* spp. depends on the use of highly specialized, urea-rich media supplemented with serum and cholesterol, such as 10B broth and A8 agar, and incubation under a 5%–10% CO_2_ enriched atmosphere is required to optimize their growth. In liquid culture systems, such as 10B broth (Shepard’s medium), microbial growth is detected through the activity of specific urease, which increases the alkalinity of the medium and induces a color change. For morphological identification, solid media such as A8 or A7 agar are employed to visualize the characteristic brown and granular colonies resulting from the precipitation of metallic oxides following urea hydrolysis. Rapid commercial systems enable simultaneous identification and antimicrobial susceptibility testing. However, despite their practical utility, these platforms present important limitations when compared with reference methods (5).

Molecular-based diagnostic methods have emerged as highly sensitive and specific tools for the detection of *Ureaplasma* spp. in genital specimens. PCR-based assays, including species-specific real-time and multiplex syndromic panels, enable rapid identification directly from clinical samples, even in cases with low bacterial loads or prior antimicrobial exposure (6, 7). Moreover, qPCR provides valuable information suggesting the differentiation between colonization and active infection (8). Diagnostic techniques are summarized in Table 1.

**TABLE 1:** Comparative diagnostic techniques for *Ureaplasma* species infections

Diagnostic technique		Advantages	Limitations
Culture methods	Conventional (e.g., blood agar)	Accessible in most clinical laboratory settings	Clinically ineffective: specific growth requirements (urea and cholesterol)
	Specialized culture (10B broth, BTB broth, A7/A8 agar, commercial systems)	Allows antimicrobial susceptibility testing (AST)irm, cost-effective method available (2)	Slow-growing (2)
Direct microscopy	Gram	Accessible in most clinical laboratory settings	Clinically ineffective: these organisms lack a peptidoglycan cell wall, making them invisible
	Dienes, Acridine Orange, Giemsa, May–Grünwald–Giemsa	Low cost, simple, useful for colony visualization (Dienes), high sensitivity for acridine orange stain	Low sensitivity and specificity, limited value in direct specimens (2)
Molecular methods	Universal PCR (16S rRNA gene amplification) and Sanger sequencing	High sensitivity, broad pathogen detection (2), culture-independent	No information regarding antimicrobial resistance unless specific genes (e.g., *tetM*) are targeted (9). Discrimination between *Ureaplasma* species was not possible unless the entire gene or specific discriminatory regions were sequenced (10)
	Species-specific PCR, multiplex PCR targeting *Ureaplasma* spp.	High sensitivity and specificity, rapid, species differentiation (Ureasa gen), quantification, culture-independent	Impossibility of distinguishing between infection and colonization in some samples
Whole-genome sequencing (WGS)	WGS, Shotgun metagenomics (metagenomic next-generation sequencing [mNGS])	High sensitivity and specificity, detection of resistance genes (2), culture-independent	High cost, risk of detecting commensal microorganisms, complex bioinformatics (2)

*Ureaplasma* spp. can be found as a colonizer of the genitourinary tract with colonization rates in healthy adults reaching up to 80% (3). When localized pathogenicity occurs, they are primarily identified as causative agents of non-gonococcal urethritis in men, and cervicitis or pelvic inflammatory disease in women. However, distinguishing the role of *Ureaplasma* spp. and other mycoplasmas as colonizers or true agents of infection remains a subject of ongoing debate. Furthermore, their role in reproductive health is significant; localized *Ureaplasma* species infection is closely linked to chorioamnionitis, spontaneous abortion, and preterm labor (11). Extragenital infection is rare and often underdiagnosed and can occur after hematogenous dissemination to extragenital sites when the host’s humoral barriers are compromised (12, 13) or following the disruption of anatomical barriers during surgery (14).

Detection of *Ureaplasma* spp. in sterile invasive samples, such as those from surgical sites or normally non-colonized anatomical locations, should be considered indicative of true infection rather than colonization. Diagnosis is often delayed due to low clinical suspicion and typically relies on molecular methods, such as universal 16S rRNA gene PCR followed by sequencing.

*Ureaplasma* species exhibit a urease-mediated metabolic activity that generates energy through urea hydrolysis into ammonia and carbon dioxide. In invasive infections, this process may lead to toxic hyperammonemia, a characteristic of disseminated *Ureaplasma* species infections, particularly in immunocompromised and transplant patients (2, 15).

Procalcitonin production is largely triggered by bacterial cell wall components, particularly peptidoglycan. In *Ureaplasma* species infections, the absence of peptidoglycan limits the activation of inflammatory pathways, resulting in a reduced procalcitonin response, even in the presence of clinically significant infections (16, 17).

Several methods are available to assess the antimicrobial susceptibility of *Ureaplasma* spp. Broth microdilution and agar dilution are considered the reference methods, allowing determination of minimum inhibitory concentrations (MICs) using specialized media such as 10B broth or A8 agar (5) according to CLSI. At present, the European Committee on Antimicrobial Susceptibility Testing (EUCAST) has not established standardized methods or clinical breakpoints for *Ureaplasma* spp. In contrast, CLSI has proposed reference testing methods; however, interpretive breakpoints remain unavailable for several antimicrobial agents, and their use is generally limited to reference laboratories due to technical complexity.

Commercial colorimetric MIC Kits are among the most widely used tools in clinical practice in Europe (e.g., Mycoplasma IST2 [bioMérieux], Myco-Well D-One [CPM SRL]). These systems provide rapid results based on colorimetric changes in the culture medium associated with bacterial growth in the presence of antimicrobial agents. Many commercial kits do not strictly adhere to Clinical and Laboratory Standards Institute (CLSI) recommendations, which may affect the comparability and standardization of susceptibility results (5).

When culture fails, mNGS or targeted PCR can identify specific mutations associated with resistance. Key targets include the *tetM* gene for tetracycline resistance and mutations in the quinolone resistance-determining regions (18).

The optimal treatment for severe or disseminated *Ureaplasma* species infections remains poorly defined. Several therapeutic regimens have been proposed, most commonly involving doxycycline in combination with a macrolide, while a fluoroquinolone may also be added in certain cases. Various studies have shown high susceptibility rates on *Ureaplasma urealyticum* strains where doxycycline and clarithromycin exhibit the lowest MIC values among all agents tested (3, 9, 18). At our institution, susceptibility testing for *Ureaplasma* spp. is not available; therefore, the patient was treated empirically with doxycycline, resulting in a prompt clinical response.

To the best of our knowledge, this represents the first documented case of surgical site infection following laminectomy attributed to *Ureaplasma urealyticum* in an immunocompetent patient. Molecular diagnostic techniques play a crucial role in achieving microbiological diagnoses in infections caused by atypical or fastidious microorganisms that are not reliably detected by conventional culture methods.

## SELF-ASSESSMENT QUESTIONS

1. Which statement best explains why beta-lactam and glycopeptide antibiotics are ineffective against *Ureaplasma* spp.?
  - These drugs cannot penetrate the bacterial outer membrane of *Ureaplasma* spp.
  - *Ureaplasma* spp. rapidly inactivate these drugs through beta-lactamase and glycopeptidase enzymes.
  - *Ureaplasma* spp. lack a peptidoglycan cell wall, so the molecular targets of these antibiotics are absent.
  - These antibiotics are actively pumped out of the cell by highly efficient efflux pumps.
2. Which of the following is the most appropriate antimicrobial therapy for *Ureaplasma* species infections?
  - Prolonged beta-lactam therapy
  - Vancomycin as monotherapy
  - Lipopeptides
  - Doxycycline
3. Which diagnostic approach is considered the most reliable for the direct detection and species-level identification of *Ureaplasma* spp. in genital specimens?
  - Gram staining followed by biochemical testing.
  - Culture on blood agar with routine incubation.
  - Species-specific PCR assays targeting conserved genes.
  - Serological testing for anti-*Ureaplasma* species antibodies.

## ANSWER TO SELF-ASSESSMENT QUESTIONS

1. Which statement best explains why beta-lactam and glycopeptide antibiotics are ineffective against *Ureaplasma* spp.?
  - These drugs cannot penetrate the bacterial outer membrane of *Ureaplasma* spp.
  - *Ureaplasma* spp. rapidly inactivate these drugs through beta-lactamase and glycopeptidase enzymes.
  - *Ureaplasma* spp. lack a peptidoglycan cell wall, so the molecular targets of these antibiotics are absent.
  - These antibiotics are actively pumped out of the cell by highly efficient efflux pumps.

Answer: c. *Ureaplasma* spp. lack a peptidoglycan cell wall, so the molecular targets of these antibiotics are absent. Beta-lactams and glycopeptides act by inhibiting synthesis or cross-linking of peptidoglycan in the bacterial cell wall; since *Ureaplasma* spp. do not possess peptidoglycan structure, these drugs have nothing to bind to and are therefore intrinsically inactive against them. Other options (reduced penetration, drug-inactivating enzymes, or efflux pumps) may explain resistance in many classic bacteria, but they do not describe the primary mechanism of intrinsic resistance in *Ureaplasma* spp.

1. Which of the following is the most appropriate antimicrobial therapy for *Ureaplasma* species infections?
  - Prolonged beta-lactam therapy
  - Vancomycin as monotherapy
  - Lipopeptides
  - Doxycycline

Answer: d. Doxycycline. Tetracyclines, particularly doxycycline, are among the most commonly recommended first-line agents for *Ureaplasma* species infections because these organisms lack a peptidoglycan cell wall and are therefore intrinsically resistant to antibiotics that target cell wall synthesis**,** including beta-lactams**,** glycopeptides (e.g., vancomycin), and lipopeptides.

In *Ureaplasma* species infections, treatment generally requires agents that inhibit protein synthesis or DNA replication, such as tetracyclines**,** macrolides**,** or fluoroquinolones, depending on the clinical setting and susceptibility profile. In adults with invasive infection, doxycycline is frequently used as a preferred option.

1. Which diagnostic approach is considered the most reliable for the direct detection and species-level identification of *Ureaplasma* spp. in genital specimens?
  - Gram staining followed by biochemical testing.
  - Culture on blood agar with routine incubation.
  - Species-specific PCR assays targeting conserved genes.
  - Serological testing for anti-*Ureaplasma* species antibodies.

Answer: c. Species-specific PCR assays targeting conserved genes.

Species-specific PCR assays provide the highest sensitivity and specificity for detecting *Ureaplasma* spp. in genital specimens. These tests target conserved and discriminatory genes, allowing accurate identification and differentiation between *Ureaplasma parvum* and *Ureaplasma urealyticum*. In contrast, Gram staining and routine culture are unreliable due to the absence of a cell wall and the fastidious growth requirements.

**TAKE HOME POINTS**

- This case highlights *Ureaplasma* spp. as rare but important causes of deep postoperative infections, especially when routine cultures remain negative despite clear clinical evidence of infection.
- *Ureaplasma* spp. lack a peptidoglycan cell wall, making them intrinsically resistant to beta-lactams and glycopeptides and explaining treatment failure with standard broad-spectrum regimens.
- Molecular methods, particularly universal 16S rRNA PCR and species-specific PCR, are crucial to achieve microbiologic diagnosis in these fastidious organisms.
- Once identified, targeted therapy with active agents such as doxycycline can lead to complete clinical, analytical, and radiologic resolution.
